# Feasibility of a Career Development Intervention for Veterans in Vocational Rehabilitation: Protocol for a Pilot Randomized Controlled Trial

**DOI:** 10.2196/47986

**Published:** 2023-06-30

**Authors:** Brian Stevenson, Taina Teravainen, Ummul Kiram Kathawalla, Margo Singer, Sarah Wilkins, Lisa Mueller, Megan Kelly, Marsha Ellison, Steven D Shirk, Shana Bakken, David Blustein

**Affiliations:** 1 Veterans Integrated Services Network 1 Mental Illness Research, Education, and Clinical Center Veterans Affairs Bedford Healthcare System Bedford, MA United States; 2 Department of Psychiatry and Population and Quantitative Health Sciences UMass Chan Medical School Worcester, MA United States; 3 Boston University Boston, MA United States; 4 VA Bedford Healthcare System Bedford, MA United States; 5 National Veterans Health Administration Vocational Rehabilitation Service Veterans Affairs Central Office Washington, DC United States; 6 Department of Counseling, Developmental, & Educational Psychology Boston College Boston, MA United States

**Keywords:** psychiatric disorders, career development, vocational rehabilitation, randomized controlled trial, transitional work, veterans, mental health, employment

## Abstract

**Background:**

Veterans with psychiatric disorders want additional career development services to support their recovery and pursuit of meaningful employment. However, no career counseling programs have been designed for this specific population. We developed the Purposeful Pathways intervention to fill this need.

**Objective:**

This study protocol aims to (1) evaluate the feasibility and acceptability of the Purposeful Pathways intervention for veterans living with psychiatric disorders and (2) explore preliminary clinical outcome data.

**Methods:**

A total of 50 veterans who are participating in transitional work vocational rehabilitation services at a Veterans Affairs hospital will be randomized to either treatment as usual or the augmented treatment condition (treatment as usual plus Purposeful Pathways). Feasibility will be assessed via recruitment rates, clinician fidelity to treatment, retention rates, and acceptability of randomization procedures. Acceptability will be assessed via client satisfaction at treatment termination using quantitative and qualitative data collection. Preliminary clinical and vocational outcomes will be assessed at baseline, 6 weeks, 12 weeks (treatment termination), and a 3-month follow-up via quantitative measures assessing vocational functioning, vocational process, and mental and physical functioning.

**Results:**

This pilot randomized controlled trial is beginning recruitment in June 2023 and is expected to continue through November 2025. Data collection is expected to be completed by February 2026, with full data analysis completed by March 2026.

**Conclusions:**

Findings from this study will provide information on the feasibility and acceptability of the Purposeful Pathways intervention, as well as secondary outcomes related to vocational functioning, vocational process, and mental and physical functioning.

**Trial Registration:**

ClinicalTrials.gov NCT04698967; https://clinicaltrials.gov/ct2/show/NCT04698967

**International Registered Report Identifier (IRRID):**

PRR1-10.2196/47986

## Introduction

### Background

Meaningful employment is consistently associated with the health and well-being of individuals living with mental health conditions [1-3], yet veterans living with psychiatric or substance use disorders often experience high levels of unemployment or underemployment [4-7]. To address this problem, the Veterans Health Administration (VHA) offers robust vocational rehabilitation programming that has helped countless veterans with psychiatric disorders obtain competitive employment. The most widely used vocational rehabilitation service within VHA is transitional work (TW) [8]. While the TW intervention varies across Veterans Affairs (VA) health care settings, it usually provides veterans with rapid access to time-limited, paid work experiences provided by VA departments, other federal agencies, or community employers through partnerships with the VA. Positions are typically housed in the VA (eg, housekeeping, grounds, kitchens, and construction) but are sometimes located in the community. To be eligible for TW, a referral needs to be made by a health care provider, and the veteran must have a vocational and clinical problem.

Research examining participation outcomes in TW has demonstrated that consumers are more likely to engage in paid activity, work more total hours, work more weeks, and earn more in total wages compared to individuals receiving state-funded vocational rehabilitation services [9]. However, research also finds that TW may not be effective at helping veterans find and secure competitive employment, even though this is a goal of the TW program [8,9]. A recent study of national TW data (N=38,199) found that only 34.8% of veterans participating in TW at a community-based job site, and 30.3% of veterans participating in TW at a VA-based site, were competitively employed at discharge [8]. Additional interventions may be needed for veterans in TW to support their employment functioning.

Ancillary vocational services such as prevocational assessment and job clubs have been shown to increase the odds (1.12 and 1.32 odds ratios, respectively) of veterans securing competitive employment when paired with a main VHA Vocational Rehabilitation Services program such as TW [8]. Given this finding, researchers recommend evaluating the impact of ancillary vocational interventions on competitive employment outcomes of veterans when paired with TW [8]. This recommendation is aligned with the voices of veterans living with psychiatric disorders who report wanting ancillary vocational services such as vocational assessment and career planning to help support their pursuit of meaningful employment and their mental health and substance use recoveries [10,11].

The overarching goal of this study is to develop and evaluate a career counseling protocol to promote vocational outcomes for veterans living with psychiatric disorders. This study consists of three phases: (1) treatment development, (2) treatment refinement through an open trial, and (3) evaluating feasibility through a pilot randomized controlled trial (RCT). The first 2 phases of this study were completed between April 2021 and February 2023 and are discussed below.

### Prior Work

During the first phase of this treatment development and evaluation project, our team conducted qualitative focus groups among veterans in TW (n=6), as well as among TW providers (n=7), to better understand the career development needs of veterans engaged in vocational rehabilitation services [12]. An advisory panel of practitioners, researchers, academic scholars, and administrators with expertise in vocational rehabilitation, career development, or treatment manualization (n=12) reviewed the findings from these focus groups and synthesized the results across the best available research and clinical practice. Collectively, four major treatment mechanisms emerged: (1) career exploration, (2) goal clarity, (3) job-searching skills, and (4) self-regulation skills.

A preliminary treatment manual was developed to address the 4 major treatment mechanisms identified. The preliminary manual was then reviewed by the 7 TW providers who previously participated in 1 of 2 focus groups. These providers offered suggestions for editing the manual. These suggestions were reviewed by the principal investigator (PI), a coinvestigator, and a research assistant and then used to revise the treatment manual. Modifications included developing additional handouts and worksheets for veterans to complete during treatment, offering more clinical case examples within the manual, and using more inclusive language.

During the second phase of our project, we conducted an open trial in which 10 veterans in TW received the newly developed career development intervention. The PI provided the treatment and maintained process notes on each session. After study completion, veterans provided qualitative feedback to a research assistant about their experiences in the intervention. In total, 7 veterans completed this exit interview, with 3 lost to follow-up. Generally, veterans provided positive feedback about the intervention. Minor adjustments were made to the manual based on their feedback, which focused on helpful counselor qualities and approaches rather than the intervention content. The PI completed a final version of the treatment manual in consultation with 2 expert consultants who previously participated on the treatment development advisory panel.

### Description of the Purposeful Pathways Intervention

Purposeful Pathways is a manualized intervention to assist veterans in aspiring and pursuing meaningful employment. Purposeful Pathways is an individual counseling protocol delivered weekly for up to 12 sessions (50 minutes per session). The intervention is divided into three sections: (1) introductory sessions (up to 3 sessions), (2) middle sessions (up to 8 sessions), and (3) discharge planning sessions (at least 1 session). In the introductory sessions, the counselor engages the veteran in developing a therapeutic bond and collaboratively developing a treatment plan. During these sessions, the counselor focuses on introductions, reviewing the context of the treatment, establishing the treatment orientation, providing psychoeducation on career development, gathering a comprehensive vocational history, exploring the veteran’s desired vocational outcomes, and collaborating on a shared understanding of the goals of their work together. In the middle sessions, the counselor and client work on 1 or all 4 of the following: career development tasks, career exploration, goal clarity, job-searching skills, and self-regulation skills. The choice of interventions is collaboratively decided upon between the counselor and veteran based on the veteran’s needs and preferences. The counselor uses cognitive behavioral therapy and motivational interviewing strategies throughout the implementation of the various interventions, such as open-ended questions, affirmations, reflections, summaries, and motivating behavior change. Career exploration includes identifying the veteran’s preferences, connecting the veteran’s preferences to the world of work, identifying needed occupational accommodations, and working through indecision. Goal clarity includes developing a visual goals map (ie, developing a plan for achieving one’s occupational goals). Job-searching skills include job-searching strategies, resume writing, and interviewing skill-building. Self-regulation skills include recognizing distractions, opportunities, and roadblocks to reaching employment goals, skills for sustaining career development over time, immediate coping skills for managing distress during recovery, and developing a vocational coping plan (ie, a plan for managing distress on a worksite). The final section of the manual, the Discharge Planning section, focuses on helping a veteran synthesize what they have learned in therapy and planning for their recovery once therapy ends. Discharge planning includes reviewing therapist and veteran perceived progress made toward goals, planning for booster sessions, and facilitating warm handoffs to additional treatment, as needed.

### This Study

This protocol represents the third and final phase of this treatment development and evaluation study. This third phase aims to (1) evaluate the feasibility and acceptability of the Purposeful Pathways intervention among veterans living with psychiatric disorders and (2) explore preliminary clinical outcome data. To accomplish this aim, we will conduct a pilot RCT comparing Purposeful Pathways to a treatment as usual (TAU) control condition with a 3-month follow-up. This study has received approval from a local VA Institutional Review Board, and the protocol has been registered with clinical trials and will begin recruitment in June 2023.

## Methods

### Recruitment

In total, 50 veterans participating in a VHA TW vocational rehabilitation program will be recruited for this study. Inclusion criteria will include (1) eligible for and planning to enroll in TW, or within 16 weeks of having enrolled in TW; (2) the presence of a current psychiatric disorder, such as posttraumatic stress disorder, mood disorders (eg, bipolar disorders and major depression), anxiety-related disorders (eg, generalized anxiety disorder, panic disorder, agoraphobia, and obsessive compulsive disorder), psychotic disorders (eg, schizophrenia and schizoaffective disorder), substance use disorders (eg, alcohol, opioid, cannabis, cocaine, sedative, hypnotic or anxiolytic, and other stimulants), which is indicated by an International Classification of Diseases-10 diagnosis encounter in the participants’ VA medical record within the last 6 months, inputted by a licensed mental health provider (psychiatrist, psychologist, licensed clinical social worker, and psychiatric nurse practitioner); (3) self-identification as either unemployed, underemployed (self-report of falling into 1 of 5 categories: overeducated, job-education mismatch, skill underuse, being involuntarily engaged in part-time, temporary, or intermittent employment [ie, hours underemployed], or earning wages in a job that are 20% less compared to a previous job, or for recent college graduates, earning wages that are 20% less compared to graduating peers of similar education or training [ie, pay underemployment] [13]), or self-report that they are employed but functioning poorly at work as a result of mental illness or substance use; (4) self-identification as not being on leave from a job for which there are arrangements to return to their job following the completion of TW; (5) having plans to work for 3 years beyond the end of this study; and (6) competency to provide written informed consent.

Exclusion criteria will include (1) acute suicide or homicide risk requiring treatment focused on safety to self or others based on self-report of current and active thoughts of harm to self or others with intent and plan that requires crisis stabilization in an acute psychiatric unit; (2) being not otherwise eligible for TW services; (3) current enrollment in compensated work therapy supported employment services; (4) Symptom Checklist-6 [14] score greater than 26; (5) Veterans RAND-12 physical component score [15] less than 12, and Veterans RAND-12 mental composite score [15] less than 8; (6) improbability that participant will complete the study for the following reasons: expected deployment, expected incarceration, expected long-term hospitalization, or expected relocation from the vicinity of the participating VA during the study period; and (7) unwillingness to engage in a weekly career development intervention.

**Figure 1:**
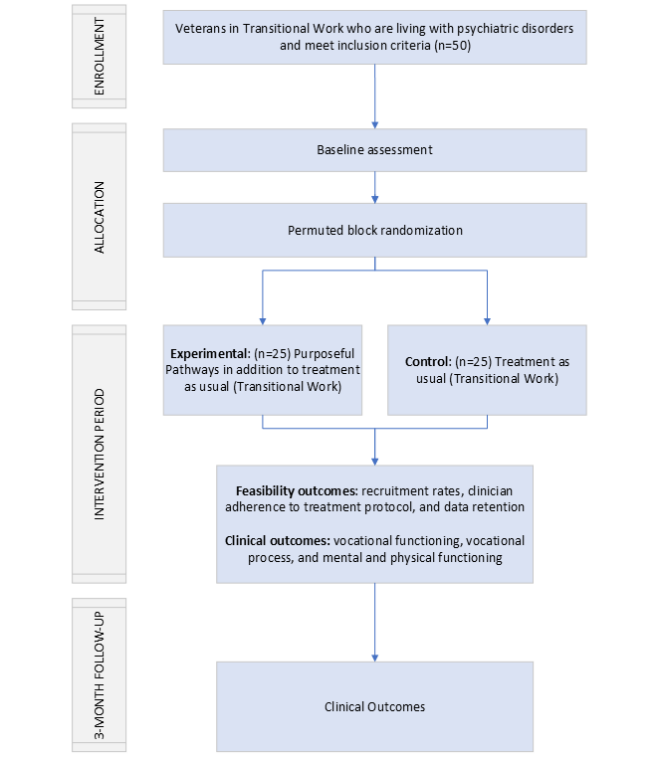
CONSORT (Consolidated Standards of Reporting Trials) diagram of the work trial.

Veterans who are eligible and complete the informed consent procedures will be randomized to 1 of 2 study arms: the treatment condition (n=25) or the control condition (n=25). An overview of the design of this pilot RCT is presented in Figure 1.

### Randomization Procedures

To ensure equal distribution across the intervention and control conditions, a permuted block randomization method will be used. Randomization will use computer-generated numbered lists, one for each condition (treatment and control). The lists will be generated through a block design where the block sizes are randomly selected among block sizes of either 4 or 6. There will be 5 rounds of each block size to randomize the 50 veterans into either the treatment or control condition. With this approach, every study arm assignment on the randomization list will consist of an even number of treatment or control assignments in random order. This approach has been discussed by previous researchers as guaranteeing balance in the 2 conditions while also ensuring that researchers are unable to predict the treatment sequence as they will not know if the block size is 4 or 6 [16]. This procedure will result in 25 participants per condition, which is in line with the recommendations of Rounsaville and colleagues [17], who suggest that each treatment condition in a stage 1b clinical trial should have at least 15 subjects. Subjects randomized to the treatment group will receive the Purposeful Pathways intervention in addition to TAU. Subjects randomized to the control group will only receive TAU.

### Participation in TAU

Both the experimental and the control condition will participate in TAU through TW. Upon admission into TW, veterans are quickly placed into a paid work setting, either at the VA hospital or in the community. There are minimal formal assessment or other vocational services prior to a veteran being placed into a worksite. TW program staff, which can range from peer support staff to master’s-trained vocational counselors, provide some on-site and off-site job coaching to help veterans maintain their TW placement, in addition to some guidance on competitive job searching. TW program staff tend to have caseloads of 25-40 veterans [9].

### Experimental Intervention

Veterans randomized to the experimental condition will augment their experience in TW with the Purposeful Pathways intervention. This intervention will be delivered to the veteran by a master’s-level mental health clinician. This intervention will be delivered on an individual basis for up to 12 sessions (depending on the unique needs and experiences of the veteran-client). The intervention has been designed to be delivered face-to-face or through web-based telehealth platforms, depending on the preferences of the veteran-client.

### Therapist Recruitment and Training

The PI will recruit and train at least 4 master’s- or doctoral-level mental health clinicians in the delivery of Purposeful Pathways (2 clinicians per 15 months). Clinicians will have specialized training in clinical social work, counseling, or clinical psychology. Before implementing Purposeful Pathways, therapists will participate in a 6-hour training workshop held over 2 or 3 days. The training workshop will be facilitated by the PI of the study.

### Therapist Adherence

There will be multiple fidelity checks to ensure treatment adherence among study clinicians. After completing the initial 6-hour training workshop, study clinicians will be required to attend weekly group consultations with the PI (1 hour per week) for the first 3 months of providing treatment to address technical issues in the provision of the manual. Additionally, all Purposeful Pathways sessions will be video recorded, and these sessions will be reviewed and rated (on a variable time schedule) for treatment adherence by 2 staff researchers using a fidelity checklist system, which includes both process and content variables necessary for treatment. Once a session is rated, researchers will provide in-person, individual feedback to clinicians.

### Assessment Measures and Timeline

A variety of clinical outcomes will be assessed at baseline, 6 weeks, 12 weeks (treatment termination), and at a 3-month follow-up. Subjects will be compensated US $40 for completing the baseline assessment, US $40 for the completing assessment at week 6, US $80 for completing the assessment and exit interview at treatment termination or week 12, and US $40 for completing the assessment at a 3-month follow-up for a possible total compensation of US $200. Table 1 provides an overview of the administration schedule for the planned measures. Descriptions of each measure are provided after Table 1.

A demographic questionnaire will be used at the screening to gather basic demographic information. Information gathered will include age, gender, race, level of education, service-connected percentage, service-connected condition, receipt of disability pensions, housing stability, and history of substance abuse.

A vocational functioning questionnaire will gather common employment outcome information. The following employment outcomes will be assessed: (1) the mean number of jobs acquired, mean duration of each job acquired, mean pay rate, and total pay per job; (2) the total weeks worked and total hours worked during the follow-up period (if relevant); and (3) the total earned income.

**Table 1:** Timeline of measures.

Measure	First session	6 weeks	12 weeks	3 months
Demographic questionnaire	✓			
Vocational functioning questionnaire	✓	✓	✓	✓
Confidence and importance rulers	✓	✓	✓	✓
Job-Search Intensity Scale	✓	✓	✓	✓
Career Adapt-Abilities Scale	✓	✓	✓	✓
Vocational Identity Scale	✓	✓	✓	✓
Decent Work Scale	✓	✓	✓	✓
Work Needs Satisfaction Scale	✓	✓	✓	✓
Quality of Life Enjoyment and Satisfaction Questionnaire	✓	✓	✓	✓
Sheehan Disability Scale	✓	✓	✓	✓
Client Satisfaction Questionnaire-8			✓	
Qualitative exit interview			✓	

Confidence and importance rulers are common tools used in motivational interviewing and cognitive behavioral therapy protocols to assess levels of confidence and importance related to a specific goal. For the purposes of this study, the specific goal would be to “find meaningful employment.” On a scale of 1-10, participants will be asked to rate their level of confidence from 0 (“not at all confident”) to 10 (“extremely confident”) and the level of importance to them from 0 (“not at all important”) to 10 (“extremely important”) to find meaningful employment. Evidence of reliability and validity has been documented by others [18].

The Job Search Intensity Scale [19] will be used to assess the level of time one spends on various job-search activities. This 9-item measure asks respondents about the following job-search activities: preparing or revising resumes, reading classified or help wanted advertisements, looking for jobs on the internet, talking with friends or relatives about job leads, speaking with previous employers or business acquaintances about job leads, contacting employment agencies, making inquiries to prospective employers, sending out application letters, and preparing and going on job interviews. Responses to each activity are measured on a 5-point Likert-type scale to assess the amount of time spent on each activity. Possible scores for each item range from 1 (“no time at all”) to 5 (“very much time”). A previous study found this measure to be valid and reliable [19].

The Career Adapt-Abilities Scale [20] will be used to measure one’s level of career adaptability, a commonly used measure of career readiness. This is a 24-item measure that is grouped into 4 different subscales that capture total adaptability along with 4 dimensions—concern, control, curiosity, and confidence. Respondents are asked to rate how strongly they have developed each of these strengths using a 5-point Likert-type scale. Responses range from 5 (“strongest”) to 1 (“not strong”). This is a widely used measure in the fields of vocational psychology and career development, with evidence of good validity and reliability [20].

The Vocational Identity Scale (VIS) [21] will measure the extent to which an individual possesses a clear and stable understanding of his or her goals, interests, and talents as related to vocational decisions. The VIS consists of 18 true or false items that ask respondents to identify whether the statement is mostly “true” or mostly “false.” A respondent’s total number of “true” responses makes up their final score (range 0-18), with higher scores indicating greater career indecision and lower scores indicating more crystallized vocational decidedness. The VIS is extensively used in vocational psychology literature and has undergone extensive psychometric analysis that has demonstrated validity and reliability as a measure of career indecision [22] and has been used in other studies among individuals with psychiatric disorders [23].

The Decent Work Scale [24] will be used to assess the quality of one’s job with respect to (1) physically and interpersonally safe working conditions, (2) access to health care, (3) adequate compensation, (4) hours that allow for free time and rest, and (5) organizational values that complement family and social values. The scale is comprised of 5 subscales (based on the factors of decent work just described) and a total scale score. This self-report measure consists of 15 items on a 7-point Likert scale, ranging from “strongly disagree” to “strongly agree” with higher scores indicating greater levels of decent work. The Decent Work Scale has evidence of reliability and validity among employed adults [24].

The Work Needs Satisfaction Survey [25] will be used to measure one’s satisfaction with their employment in terms of meeting their survival or power, social connection, and self-determination needs. This is a 20-item measure that prompts respondents to rate an item on a 7-point scale ranging from “strongly disagree” to “strongly agree.” Evidence of validity and reliability was demonstrated by the scale developers [25].

The Quality of Life Enjoyment and Satisfaction Questionnaire–Short Form [26] will be used to measure general psychiatric functioning. The Quality of Life Enjoyment and Satisfaction Questionnaire–Short Form is a 16-item self-report measure of perceived functioning and is one of the most widely used outcome measures in psychiatric research. Reliability and validity of this measure have been extensively documented.

The Sheehan Disability Scale [27] will measure the level of functional impairment due to mental health and medical impairments. This self-report measure asks raters to how much their work or school, social life, and home life or family responsibilities are impaired by mental and medical symptoms. This is a 5-item measure using a 10-point visual analog scale. The Sheehan Disability Scale has received extensive psychometric testing for validity and reliability [27].

The Client Satisfaction Questionnaire–8 (CSQ-8) [28] will be used to measure satisfaction, perceived quality, and effectiveness of the treatment. This self-report questionnaire consists of 8 items, scored 1-4, with higher scores indicating higher satisfaction with treatment. The CSQ-8 has been used in mental health and other health centers and has acceptable internal consistency (α=.83 to .93) [28].

In addition to the above measures, subjects will complete a semistructured 30-minute exit interview at treatment termination or 12 weeks. This semistructured interview will be conducted by a researcher not involved in the delivery of treatment. These interviews will be recorded and transcribed for later analysis. The following content areas will be assessed through this interview: (1) general thoughts or experiences with treatment, (2) perceived benefits of participating in treatment, (3) specific changes and outcomes obtained because of participating in treatment, (4) perceived limitations of the treatment, and (5) recommendations for enhancement and improvement of services. We will also ask open-ended questions about the usability of measures and assessments.

### Data Analysis

Primary analyses will involve the examination of the feasibility and acceptability. Feasibility will be verified by adequate (1) recruitment rates (ie, 2-3 veterans per month), (2) >90% fidelity of clinician adherence to the treatment protocol, and (3) data retention (ie, ≥70% of participants who attend at least 6 sessions of Purposeful Pathways. An acceptable level of attrition will be ˂30% in each condition). We will also examine the willingness of veterans to be randomized with a goal of 80% of those approached who agree to randomization, the number of referrals from clinicians, ease of recruitment based on the average number of contacts needed to get consent, and the number of eligible participants from the pool of potential participants. We will compare veteran acceptability between groups by conducting an independent samples *t* test on the CSQ-8 [28]. We will compare attrition rates (<30% vs >30%) between the 2 groups using a chi-square and logistic regression analysis.

Qualitative data gathered during the exit interviews will be analyzed using the Hamilton Rapid Turn-around Analytic Approach [29]. This technique is suited to short-term projects using targeted interview guides. It improves on past rapid procedures through its systematic, rigorous approach, yielding results that are slightly less nuanced but more efficient. The PI will develop an analytic template organized by the topic areas from the semistructured interview guiding qualitative interviews. The PI and at least 2 other researchers will apply the template to summarize 2 interviews to test the usability and relevance of the template to the transcripts. The research team will then meet to establish consistency in how the template is used to summarize the transcripts. From here, all focus groups will be individually summarized (each focus group will be analyzed by at least 2 researchers to ensure reliability). The PI will then transfer all the summary points into a master matrix (respondent × topic area). Next, the PI will condense the summaries in the master matrix by identifying and organizing recurring themes. The other members of the research team will review the condensed matrix to the original master matrix to confirm all central themes have been identified and ensure consensus.

Finally, we will examine differences in our clinical and vocational measures to identify empirical targets for further refinement of the treatment and inform the design of future, fully powered efficacy trials. Based on the intent-to-treat principle, all participants randomized to treatment will be included in analyses. We will run descriptive statistics as well as internal reliability (Cronbach α) for all measures. We will additionally examine the Pearson *r* to look at relationships between variables, Cohen *d* for any continuous measures, and Cramer *V* for any categorical measures to look at effect size. We will also look at individual trajectories of response to gain better insight into which participants do particularly well (or poorly) in the 2 interventions. Effect sizes and 95% CIs will be used as only 1 element to estimate the power and sample size needed for future efficacy trials.

### Ethical Considerations

All phases of this study were approved by the institutional review board of the VA Bedford Health Care System Research and Development Committee in Bedford, Massachusetts, United States, with initial approval granted in February 2021. This pilot trial was registered at ClinicalTrials.gov (NCT04698967).

## Results

This study was funded in October 2020, with a project start date of April 2021. Participant recruitment for this phase 3 pilot RCT began in June 2023. We anticipate study recruitment to be completed by November 2025 and primary data analysis completed by March 2026.

## Discussion

### Anticipated Findings

This pilot randomized control trial will provide feasibility and acceptability data for the Purposeful Pathways intervention, a manualized intervention to assist veterans in aspiring and pursuing meaningful employment. This pilot RCT will also provide data regarding the feasibility of the protocol procedures, which can help inform future efficacy trials. Finally, the findings will produce preliminary data regarding significant effects on specific outcome measures, which will inform further refinement of the treatment protocol and the development of future efficacy trials.

### Conclusions

Meaningful employment is associated with the health and well-being of individuals living with psychiatric disorders [1-3]; however, the benefits gained from meaningful employment extend to few veterans, who as a group face high levels of un- and underemployment [4-7]. While previous research has shown that an integration of services like vocational assessment along with a primary VHA vocational rehabilitation program improves the likelihood of veterans obtaining competitive employment [8], there remains a gap in our knowledge of evidence-based approaches to such services. This pilot study addresses this critical need for evidence-based career counseling and development services for veterans living with psychiatric disorders. The trial will serve as the first step in testing whether enhancing VA vocational rehabilitation services with a concurrent, targeted career counseling and development protocol is feasible and acceptable for veterans with psychiatric disorders, as well as initial estimates of potential benefits.

## Appendix

Multimedia Appendix 1 Peer-review report by the Career Development Program - Panel II - Rehabilitation Research and Development Parent IRG (RRD9) - Office of Research & Development (National Institutes of Health, USA).

## Abbreviations

CSQ-8: Client Satisfaction Questioannaire-8
PI: principal investigator
RCT: randomized controlled trial
TAU: treatment as usual
TW: transitional work
VA: Veterans Affairs
VHA: Veterans Health Administration
VIS: Vocational Identity Scale

## Data Availability

Owing to privacy and ethical considerations, final data sets underlying publications resulting from this research will not be publicly shared outside the Department of Veterans Affairs. On request and with principal investigator (BS) permission, a deidentified, anonymized data set will be created and shared pursuant to a data use agreement, appropriately limiting the use of the data set and prohibiting the recipient from identifying or reidentifying (or taking steps to identify or reidentify) any individual whose data are included in the data set.
