# Supplementary material for: Feasibility of a Career Development Intervention for Veterans in Vocational Rehabilitation: Protocol for a Pilot Randomized Controlled Trial
Source: JMIR Res Protoc. 2023 Jun 30;12:e47986. doi: 10.2196/47986 (PMC10365634; doi:10.2196/47986)
Supplement: Multimedia Appendix 1 [file resprot_v12i1e47986_app1.pdf]

SUMMARY STATEMENT

PROGRAM CONTACT:	(Privileged Communication)	*Release Date:*	09/08/2020
		Revised Date:	

Application Number: 1 IK2 RX003401-01A2

Principal Investigator — *Formerly:* 1IK2RX003401-01A1

STEVENSON, BRIAN

Applicant Organization: EDITH NOURSE ROGERS MEMORIAL VETERANS HOSPITAL

Review Group: RRD9
Career Development Program - Panel II
RRD9

Meeting Date: 08/12/2020 — *RFA/PA:* RX20-006
Council: OCT 2020
Requested Start: 01/01/2021

Project Title: Improving Vocational Outcomes of Veterans with Psychiatric Disorders: Career Counseling & Development
SRG Action: Impact Score:113

Human Subjects: 30-Human subjects involved - Certified, no SRG concerns
Animal Subjects: 10-No live vertebrate animals involved for competing appl.
Gender: 1A-Both genders, scientifically acceptable
Minority: 1A-Minorities and non-minorities, scientifically acceptable
Age: 1A-Children, Adults, Older Adults, scientifically acceptable
Clinical Research - not NIH-defined Phase III Trial

Project Year	Direct Costs Requested
1	157,936
2	164,519
3	171,594
4	175,887
5	176,402
TOTAL	846,338

ADMINISTRATIVE BUDGET NOTE: The budget shown is the requested budget and has not been adjusted to reflect any recommendations made by reviewers. If an award is planned, the costs will be calculated by VA Office of Research and Development (ORD) staff based on the recommendations outlined in the BUDGET COMMENT section and any relevant ORD service-specific limitations.

SUMMARY OF DISCUSSION:
A Subcommittee of the Rehabilitation Research and Development Scientific Merit Review Board met in Plenary Session and reviewed the above proposal considering all internal and external reviews. This document summarizes the major points of the discussion concerning the proposed project. In any further development of this project, the investigator should consider carefully all the issues reflected in this Summary of Discussion as well as the more detailed comments in the individual critiques.

GENERAL COMMENTS:
The applicant has been highly responsive to prior reviews, and has produced an extremely compelling application. Discussion focused on strengths of the application, and did include a few recommendations.

For the research portion of the proposal, the investigator proposes to use video for fidelity checks that will be developed over the course of the CDA. However, unless visual coding is required, Dr Stevenson may want to revisit the choice of video due to confidentiality issues. Many Veterans may be leery of being videotaped, and may want the option to restrict recording to audio. In addition, video recording can result in substantial audio quality problems due to location of camera microphone which can hinder fidelity monitoring of in-session conversation. If video is not needed, then audio recording should be offered and will likely be acceptable to the Veteran give the prevalence of audiotaping in VA treatment provision and research.

Second, this intervention has potential to effect improvement in a variety of areas, and so inclusion of a measure of general improvement in mental health functioning such as the Sheehan Disability Scale or other frequently used general mental health indices would allow further comparison of this intervention's effects with other, more focused interventions.

SUGGESTIONS:
The reviewers felt that, as mentioned in prior reviews, it would serve the investigator well to clearly distinguish this intervention from existing interventions in terms of description and implementation.

COMMENTS ON THE BUDGET:
None.

DESCRIPTION (provided by applicant):
The Veterans Health Administration (VHA) offers robust vocational programming that have helped countless Veterans obtain competitive employment; however, these services are not uniformly effective as recent data suggests that only 35 to 43% of Veterans are competitively employed at time of discharge. For those who become competitively employed, job tenure may be brief, which is often attenuated by underemployment or poor person-job fit. Moreover, only 3.5% of Veterans experiencing vocational problems engage in vocational services offered by the VHA. On average, it takes Veterans more than four years to utilize vocational services. These Veterans are at high risk of acquiring multiple functional losses and developing chronic disabilities as their vocational needs go unmet for years. Research suggests that intrinsic factors like lacking clear vocational goals, perceiving barriers to employment, and negative beliefs about one's ability to work contribute to low engagement, outcomes, and tenure of some consumers of vocational rehabilitation services. Thus, the VA may be able to improve vocational engagement, outcomes, and tenure of Veterans with psychiatric disorders by enhancing vocational services with added interventions targeting unhelpful psychological factors. Career counseling and development services have been shown to be effective in helping civilian populations clarify vocational goals and identity, enhance vocational self-efficacy, and increase proactive vocational behaviors in the face of obstacles. Additionally, career counseling and

development services help facilitate greater “match” between a person and their job, and person-job match is a key determinant of long-term career tenure among individual with psychiatric disorders. The researchers of this project propose a three-aim study to develop a career counseling and development intervention for Veterans with psychiatric disorders (CCD-V). The first aim will focus on the design and development of the CCD-V intervention with Veteran and provider input (n=16). The second aim will pilot test the intervention in an open trial (n=10) to gather Veteran input on the initial intervention. The third and final aim will consist of a feasibility pilot randomized controlled trial (n=50) to examine acceptability and feasibility outcomes and to explore the impact of the CCD-V intervention in terms of functional improvement and other vocational outcomes. The proposed CCD-V intervention will consist of approximately eight individual sessions that will be offered concurrently with existing VHA vocational rehabilitation services, (e.g., transitional work experience [TWE]).The final product of this study is to produce a manualized CCD-V intervention, and corresponding fidelity monitoring checklist, to be tested later in a larger efficacy trial.

PUBLIC HEALTH RELEVANCE:
Meaningful employment is consistently associated with the health and well-being of individuals living with psychiatric disorders, yet few Veterans reap this benefit due to high levels of un- and underemployment. Thus, there is a critical need for interventions to help Veterans with psychiatric disorders find and maintain meaningful employment. The Veterans Health Administration (VHA) is committed to improving employment outcomes among Veterans and is particularly interested in meaningful employment as important for suicide prevention. This research proposal seeks to enhance vocational rehabilitation services by developing and testing an evidence-informed career counseling and development protocol. This work will fill a substantial gap in VHA vocational services as empirically informed career counseling and development interventions for Veterans living with psychiatric disorders does not currently exist.

CRITIQUE 1

Applicant: The applicant continues to be an excellent applicant for the CDA program. Dr. Stevenson will develop and conduct a small feasibility based RCT of a VA based vocational intervention that addresses psychological factors that may impede successful employment. The VA has transitional and supported work employment programs, but the applicant’s program provides relatively more focus on addressing mental health aspects of vocational success. As was the case during the last two submissions, he is currently conducting both clinical and research work in this area, with vocational leaders of the VA. He remains well positioned to contribute to, and remains committed to a career in the VA in this area. As mentioned in the prior review, the applicant’s background shows a history of work in the area of the proposed study, meshing well with the refined proposed training plan. This is a strength.

As mentioned in prior reviews, the applicant’s connection to the VA begins in graduate school, with his first position after graduate school with the Bedford VA in 2015. Since then, he has worked in the area of his proposed study at the VA. Since the first submission, he has increased his publication track record with his mentors each round. Of note, he is already involved in program innovation and evaluation, having developed a MIRECC funded vocational program in VA space with his primary mentor. He remains an excellent applicant for the CDA mechanism.

Mentor(s): The mentorship team remains unchanged from the prior application, with additional clarification given to how primary mentors and the applicant will interact. Once again, all prior comments regarding the team remain relevant and accurate: This team is longstanding and successful

in terms of publication, grant acquisition, and grant implementation. The primary mentor, Dr. Lisa Mueller is a leader in the area of vocational rehabilitation in the VA, and notably, has both developed best practices programs with the applicant (e.g., their current Vocational Evaluation Center at the Bedford VA, on which they both offering clinical services and collecting research data) and engaged in research programs with the applicant (e.g., assisted applicant in obtaining pilot award funding through the VISN 1 Mental illness research, education, and clinical center (MIRECC); collaborating with the applicant on Dr. Sandra Resnick's national CWT dataset analysis) and other research endeavors.

Dr. Kelly is a co-mentor and has also worked with the applicant, and was instrumental in helping the applicant with his MIRECC award. She is a funded researcher with expertise in mental health treatment. Dr Bakken is a national level vocational rehabilitation specialist and will assure that programs developed by the applicant are VA ready and relevant. Dr. Ellison is a research health scientist and associate director of reintegration services at the Bedford VA, and is thus both an excellent clinical and research mentor in this area. Dr. Blustein is a co-mentor and an expert in the theory of career development among marginalized populations, and will directly assist in intervention development. In addition, the applicant has also already worked with the consultant, Dr. Uma Millner to examine relationships between career development variables and employment behaviors. Finally, the applicant and the consultant have participated in Boston University's Opening Doors project, a vocational rehabilitation project upon which much of the applicant's intervention is based. The mentors' letters of support are very strong.

Overall, it is clear that the mentorship team is strong, committed to the applicant, and engaged in research and clinical work relevant to the applicant, the applicant's area of proposed research, and the VA. The mentoring team is large, but there is a clear distinction between primary and secondary mentors, and a nice integration of roles across the training plan.

Training Program: There were very few questions regarding the revised training plan submitted the last round as the applicant added needed detail regarding mentorship interaction, research goals, coursework, and logistics. The training program is well matched to the applicant's needs and the proposal's research goals and methods. As mentioned in the past, this synchrony is a strength. Needed detail has been added since the last submission. A summary list of gaps to be addressed by training is complemented by clear and pragmatic attention to the logistics associated with addressing these gaps. information on his statistical training and future training intentions are given and justified. Clear schedules of training and mentorship events have been given and the details of the proposed learning plan are clear. All prior concerns have been addressed.

Scientific Merit:

Significance: As mentioned, the applicant proposes to create and preliminarily evaluate feasibility of a vocational intervention to address factors related to employment engagement that have been found to predict vocational success, including negative beliefs about one's ability to work, lack of clear vocational goals, perceived barriers to employment, etc., among individuals with severe mental illness The applicant provides a clear conceptual linkage of that final characteristic to the intervention.

Approach: Three phases of the research plan correspond to the 3 aims and are clearly presented, and the content of the treatment and the origination of data to drive these decisions has been greatly specified. In the first aim the applicant will develop a career counseling treatment manual for Veterans with psychiatric disorders and a fidelity monitoring checklist (n=16). Specifically, through focus groups with Veterans (and responsive to prior reviews, a good mix is used:…half who obtain employment and half who were not successful) and providers (from outside his VA, also responsive to reviews), the

manual will be developed using existing programs as a guiding starting point for discussion. Using such a variety of Veterans and outside providers will give rich information, particularly from Veterans for whom programs were not successful. Extensive detail is given as to how these qualitative data will be collected and analyzed, and then used to refine the treatment itself. Specifically, similar interview guides will be used for clinicians and Veterans who do and do not succeed in obtaining employment, which will result in focused, albeit rich information directly relevant to intervention modification. Clarification of how the proposed treatment differs from the opening doors program has also been provided.

In aim 2, the manual will be pilot tested with 10 Veterans. With this revision, much more information is given as to how exit interviews and other data that could inform refinement will be used at this stage to refine the treatment, which is now encouraging.

In aim 3, a small RCT will be conducted with 50 Veterans comparing the manualized treatment to a control group. An iterative approach to manual and treatment development will be used, and, as mentioned previously that there are several ‘onramps’ for incorporating information from clinicians and Veterans for intervention refinement. The RCT sample size of 50 is appropriate.

Addressing concerns about comparator heterogeneity, the new intervention will be compared to the most popular VA vocational program, TWE transitional work experience. Excellent justification is given for this selection (rather than simply making the selection by fiat). Measures selection is well done and complete, and a more global measure of psychological functioning/quality of life are included. With this revision, much more detail is given regarding how qualitative data will be obtained and how they will be used to inform intervention refinement.

A post treatment and a three-month follow-up assessment are specified. Fidelity checks for treatment adherence are planned, and clinicians will be actual VA clinical staff. Data analyses are focused on very basic measures of feasibility, and seem appropriate.

Resources: Bedford VA resources remain unchanged. The site has ongoing research infrastructure to support this CDA applicant and affiliations with nearby academic universities among the mentoring team are strong. Moreover, some educational resources of Universities with which the mentoring team is affiliated are available to the applicant. Resources therefore seem strong.

Protection of Human Subjects: No concerns.

Inclusion of Women, Minorities, and Children: Children are appropriately excluded. Women and minorities are appropriately included.

Critique of Vertebrate Animals Section:

	Yes	No
Research with vertebrate animals?		x

Biohazards and Radioisotopes: No comment.

Additional Review Criteria (unscored)

Budget (unscored)**:** No concerns.

Data Management and Access Plan (for data sharing, unscored)**:** Data management and access seem appropriate.

Overall Strengths: Strengths remain from the original and revised applications, including a very clear conceptualization of the reciprocal relevance of work to mental health. The training plan and the research plan jibe well with the approach for intervention development, refinement, and RCT feasibility testing. The applicant has been highly responsive to reviews. The proposed work fits with the applicant's pathway of research focus and development. The research plan is logical, built on an excellent conceptual base and pilot data collected, in part, by the applicant and his mentoring team, and fits perfectly with the training plan. The training plan is on target, and mixes coursework, observation, and experiential learning.

Overall Weaknesses: The applicant has addressed all prior noted weaknesses.

CRITIQUE 2

Applicant: Dr. Stevenson is a clinical psychologist at the Bedford VA who appears sincerely committed to conducting career development research and providing career development services to a wide range of vulnerable Veteran populations. His educational background, clinical training, and research to date are well aligned with his stated goal of becoming an independent scientist-practitioner with expertise in designing and implementing interventions for Veterans living with psychiatric disorders and other psychosocial barriers, He worked with his primary mentor to develop the Vocational Evaluation Center (VEC) at his VA facility, which provides recovery-oriented career counseling and development services for Veterans; this is an impressive and relevant accomplishment. His letters of support are very strong.

Mentor(s): The application describes a robust and large mentorship team, including Dr. Lisa Mueller (primary mentor – focused on vocational development and implementation research); Dr. Megan Kelly (co-mentor – a clinical trialist with expertise in psychosocial intervention development); Dr. Shana Bakken (co-mentor – National Director of VHA Vocational Rehabilitation Services); Dr. Marsha Ellison (co-mentor – expertise in recovery and rehabilitation for persons with serious mental illness, with a focus on career services); Dr. David Blustein (co-mentor – renowned researcher in career development and psychology of working theory); and Dr. Steven Shirk (co-mentor – expertise in integrative statistical modeling to describe and understand longitudinal clinical and cognitive changes). The mentors have the requisite expertise to guide the applicant in this CDA2.

In addition, the applicant describes 5 consultants as adjunctive to his mentorship team, including Dr. Uma Millner, who has expertise in qualitative methods, and Drs. Ryan Duffy, Maureen Kenny, Blake Allen, and Kelsey Autin who all have expertise in the Psychology of Working theoretical model. Additional clarity about the distinct and complementary roles of the consultants would bolster the strength of the mentoring plan.

Training Program: Six training goals are outlined:
- Expertise in clinical trials methodology;
- Increased knowledge in advanced statistics and qualitative analysis;
- Skills in community reintegration research;
- Scientific writing and grant writing;
- Research ethics; and

- Expertise and leadership in the integration of career counseling and development and vocational rehabilitation. A variety of training experiences are proposed, including formal coursework, directed readings, discussions and meetings, site visits, and an NIH summer institute.

The proposed training activities seem largely appropriate. In addition to advanced statistics, rather than having aim 2 focus on qualitative analysis, this reviewer would reframe this aim to encompass qualitative methods more broadly (including the development and use of semi-structured interview guides and focus group prompts; sampling strategies for studies using qualitative and/or mixed methods; etc.). Though the application was revised to clarify, in response to reviewer feedback, that qualitative methods are not a statistical method, the text following this training aim still describes proposed coursework in qualitative analysis as a course in statistics.

Scientific Merit:

Significance: The proposed work has is highly significant within VA. Employment is a critical functional outcome for Veterans with psychiatric disorders; yet, this group – despite having access to robust vocational services – has significant challenges finding, securing, and maintaining employment. Adding career counseling and development services to TWE has strong potential to enhance vocational rehabilitation outcomes for vulnerable Veterans with mental illness.

Approach: Three aims are outlined:
- To develop a career counseling and development treatment manual for Veterans with psychiatric disorders (CCD-V) along with a fidelity monitoring checklist based on Veteran and provider needs assessment.
- Pilot test the manual by treating a small number of Veterans in an open trial.
- Conduct a feasibility pilot randomized controlled trial comparing CCD-V to TAU.

In general, the applicant was very responsive to prior reviews. In particular, using CCD-V as an adjunct to TWE (as opposed to TWE and SE) was a responsive and very positive change. A few suggestions:
- There could have been a bit more clarity as to if Aim 1 intended to develop a novel CCD-V intervention or rather to adapt and tailor existing interventions of this nature which have evidence in prior literature.
- The VR-12 may not be an optimal functional measure for Veterans with SMI, this is traditionally conceptualized as a HRQOL measure.
- This reviewer appreciates the appendices that are semi-structured interview guides, but investigator may consider some edits to the interview guides before they are used. There is no “grand tour” question to start either qualitative interview. Some of the nomenclature in the Veteran interview might benefit from pilot testing to ensure understanding, tailoring the interview as needed in response to this pilot testing. In addition, the qualitative interview guide for providers included some questions about content and some about implementation. The research plan does not maximally capitalize on the valuable implementation data collection that would be collected in the interview guides, so the applicant may benefit from working through this with his qualitative expert mentor.

Resources: No concerns about resources. Dr. Stevenson is embedded in a well-recognized academic environment and has the needed support for his proposed research and training plans.

Protection of Human Subjects: No concerns.

Inclusion of Women, Minorities, and Children: No concerns.

Critique of Vertebrate Animals Section: Not applicable.

	Yes	No
Research with vertebrate animals?		X

Biohazards and Radioisotopes: Not applicable.

Additional Review Criteria (unscored)

Budget (unscored)**:** No concerns.

Data Management and Access Plan (for data sharing, unscored)**:** No concerns.

Overall Strengths:
- Excellent applicant with commitment to recovery-oriented interventions in the area of vocational services.
- Very strong mentorship team.
- Important topic with strong significance for VA.

Overall Weaknesses:
- Consider an alternate functional measure to the VR-12.
- Pilot testing of the qualitative interview guides will be useful, as well as thinking about how to capitalize on the implementation data gathered from the qualitative data collection.

CRITIQUE 3

Applicant: Dr. Stevenson is a strong applicant, very committed to a career as a VA researcher. He has fostered collaborative and productive relationships with his mentors as well as incorporated himself into the services provided in the VA as well as community clinical settings. He also has established a record of publications and presentations in the area of interest (although difficult to determine exactly how many publications he has from his biosketch). These relationships and experiences will inform the development of the CCD-V intervention.

Mentor(s): This is a strong mentorship team, and the letters of support from all of them are detailed and highly complementary of the applicant. The mentors also have an extensive background in relevant clinical research that will enhance the training of the applicant.

The mentorship team also has a very strong record of both NIH and VA RR&D funding, which will provide the applicant many potential avenues for funding his independent line of research following the completion of the CDA2.

Coordination among the mentors is explicitly described, and each of the mentors have extensive experience in working with individuals early in their career, further enhancing confidence in the team.

Training Program: The training program is very detailed regarding the coursework and training that the applicant will receive in his proposed 6 training goals:
- Training Goal 1: Develop expertise in clinical trials methodology.

- Training Goal 2: Increase applicant's knowledge in advanced statistics and qualitative analysis relevant to the analyzing of treatment research.
- Training Goal 3: Improve knowledge and skill in conducting community reintegration research focused on functional outcomes.
- Training Goal 4: Strengthen scientific writing and grant writing.
- Training Goal 5: Increase expertise in research ethics.
- Training Goal 6: Strengthen applicant's expertise and leadership in the integration of career counseling and development and vocational rehabilitation.

All of the mentors have datasets and expertise, over overlapping, in these 6 areas and the ambitious training plan does appear to be feasible. Provision of tables regarding the specific aspects of the goals and timeline, as well as a proposed schedule, was very helpful to this reviewer. These skills will also inform the applicant's proposed research plan.

Scientific Merit:

Significance: The proposed intervention, CCD-V, addresses an important gap in current vocational services provided by the VA. The findings from this study will inform a Merit or R level proposal, to be written in the fourth year of the award period. This intervention could be widely disseminated to the 177 CWT programs across the nation to improve the placement and subsequent retention of employment by Veterans or all eras.

Approach: The CDA2 award will develop and evaluate a career counseling and development protocol for Veterans (CCD-V) living with psychiatric disorders. The following aims will be completed:

Aim 1. Develop a career counseling and development treatment manual for Veterans with psychiatric disorders (CCD-V) along with a fidelity monitoring checklist based on Veteran and provider needs assessment (n=16).

Aim 2. Pilot test the manual by treating a small number of Veterans (n=10) in an open trial.

Aim 3. Conduct a feasibility pilot randomized controlled trial comparing CCD-V (n=25) to treatment as usual (n=25).

3a. Evaluate feasibility and acceptability of the treatment.

3b. Evaluate the preliminary efficacy of the treatment.

Overall, the approach has been well thought out and has incorporated the suggestions of two rounds of reviewers. The shift to comparing the CCD-V to treatment as usual, rather than a time and attention control, was wise and will enhance the relevance of the findings. A time and attention control may be more appropriate for a Merit RTC.

One concern of this reviewer is that the fidelity monitoring seems under developed, and it is not clear exactly how the interventions will be delivered – using CBT? Motivational Interviewing? A didactic approach? The monitoring of the process of the sessions, as well as the content, may be important to monitor and assist in interpreting the outcomes, especially in a Veteran population. The decision to videotape sessions, rather than audiotape them, also seems unnecessarily invasive and may influence recruitment and the sessions, as well as increase the chances of a breach of confidentiality if the videos are lost or viewed by non-project individuals (as opposed to voice).

Resources: The site of the proposed research is at an excellent VA with resources to permit the training and research plan of the applicant:

- The Edith Nourse Rogers Memorial Veterans Hospital (Bedford VA).
- VISN 1 New England Mental Illness Research, Education, and Clinical Center (VISN 1 New England MIRECC).
- Social and Community Reintegration Research Program (SoCRR).
- Center for Healthcare Organization and Implementation Research (CHOIR).

The applicant is well integrated into the VA and surrounding community, which further ensures that he will take advantage of these resources.

Protection of Human Subjects: No concerns.

Inclusion of Women, Minorities, and Children: Appropriate and no concerns.

Critique of Vertebrate Animals Section:

	Yes	No
Research with vertebrate animals?		X

Biohazards and Radioisotopes: Not applicable.

Additional Review Criteria (unscored): Not applicable.

Budget (unscored)**:** No concerns; the Director of the Bedford VAHCS has committed to funding the other 25% of the applicant's salary. Budget for research costs appropriate.

Data Management and Access Plan (for data sharing, unscored)**:** No comment.

Overall Strengths: The applicant has been very thorough in addressing concerns from the previous reviewers. This has resulted in a tightly interconnected training plan and research project. This is an excellent application.

Overall Weaknesses: Minor concern regarding treatment fidelity monitoring and decision to videotape rather than audiotape.

MEETING ROSTER

Career Development Program - Panel II
Rehabilitation Research and Development Parent IRG
Office of Research & Development
RRD9

RRD9
08/12/2020

CHAIRPERSON(S)

NAYLOR, JENNIFER C, PHD
PSYCHOLOGIST
DURHAM VA MEDICAL CENTER
ASSOCIATE PROFESSOR
DEPT OF PSYCHIATRY
DUKE UNIVERSITY MEDICAL CENTER
DURHAM, NC 27705

PADGETT, LYNNE S, PHD
HEALTH PSYCHOLOGIST
DEPARTMENT OF VETERANS AFFAIRS
VETERANS HEALTH ADMINISTRATION
WASHINGTON DC VA MEDICAL CENTER
WASHINGTON, DC 20422

MEMBERS

ACIERNO, RONALD E., PHD
PSYCHOLOGIST, PTSD CLINICAL TEAM
CHARLESTON VA MEDICAL CENTER
DIRECTOR, TRAUMA AND RESILIENCE CENTER
LOUIS FAILLACE DEPARTMENT OF PSYCHIATRY
MCGOVERN MEDICAL SCHOOL AT UTHEALTH HOUSTON
HOUSTON, TX 77054

ADDISON, ODESSA, PHD *
RESEARCH HEALTH SCIENTIST
BALTIMORE VA MEDICAL CENTER
ASSISTANT PROFESSOR & DIRECTOR OF RESEARCH AFFAIRS
DEPT OF PHYSICAL THERAPY & REHABILITATION SCIENCE
UNIVERSITY OF MARYLAND, SCHOOL OF MEDICINE
BALTIMORE, MD 21201

ANTONUCCI, SHARON, PHD *
DIRECTOR
MOSSREHAB APHASIA CENTER
ELKINS PARK, PA 19027

BORSARI, BRIAN, PHD *
PSYCHOLOGIST CLINICIAN INVESTIGATOR
SAN FRANCISCO VA MEDICAL CENTER
PROFESSOR OF PSYCHIATRY
WEILL INSTITUTE FOR NEUROSCIENCES
UNIVERSITY OF CALIFORNIA, SAN FRANCISCO
SAN FRANCISCO, CA 94121

BRACH, JENNIFER S, PHD
ASSOCIATE DEAN OF FACULTY AFFAIRS AND DEVELOPMENT AND PROFESSOR
DEPARTMENT OF PHYSICAL THERAPY
SCHOOL OF HEALTH AND REHABILITATION SCIENCES
UNIVERSITY OF PITTSBURGH
PITTSBURGH, PA 15260

BRITTON, PETER C, PHD *
PSYCHOLOGIST
CANANDAIGUA VA MEDICAL CENTER
CENTER OF EXCELLENCE
CANANDAIGUA, NY 14424

CARNABY, GISELLE D., PHD *
PROFESSOR AND DIRECTOR, SWALLOWING RESEARCH LABORATORY
DEPARTMENT OF COMMUNICATION SCIENCES AND DISORDERS
COLLEGE OF MEDICINE
UNIVERSITY OF CENTRAL FLORIDA
ORLANDO, FL 32826

CLARK, DAVID J., DSC *
RESEARCH HEALTH SCIENTIST
MALCOM RANDALL VA MEDICAL CENTER
ASSOCIATE PROFESSOR
DEPARTMENT OF AGING & GERIATRIC RESEARCH
UNIVERSITY OF FLORIDA
GAINESVILLE, FL 32608

EVANS, GINA L., PHD
PSYCHOLOGIST AND INVESTIGATOR, BEHAVIORAL HEALTH AND IMPLEMENTATION PROGRAM
MICHAEL E. DEBAKEY VA MEDICAL CENTER
ASSOCIATE PROFESSOR
MENNINGER DEPT OF PSYCHIATRY & BEHAVIORAL SCIENCES
BAYLOR COLLEGE OF MEDICINE
HOUSTON, TX 77030

FAIRCHILD, JENNIFER KACI, PHD *
ASSOCIATE DIRECTOR AND FELLOWSHIP TRAINING DIRECTOR
VISN 21 MIRECC - VA PALO ALTO HEALTHCARE SYTEM
CLINICAL ASSOCIATE PROFESSOR
DEPARTMENT OF PSYCHIATRY AND BEHAVIORAL SCIENCES
STANFORD UNIVERSITY SCHOOL OF MEDICINE
PALO ALTO, CA 94304

FISHER, JONATHAN A.N., PHD
ASSISTANT PROFESSOR
DEPARTMENT OF PHYSIOLOGY
NEW YORK MEDICAL COLLEGE
VALHALLA, NY 10595

GABRIELIAN, SONYA EMI, MD *
PSYCHIATRIST AND HEALTH SERVICES RESEARCHER
VA GREATER LOS ANGELES HEALTHCARE SYSTEM
ASSISTANT PROFESSOR
DEPARTMENT OF PSYCHIATRY
UNIVERSITY OF CALIFORNIA, LOS ANGELES
LOS ANGELES, CA 90035

GARD, STEVEN A., PHD *
RESEARCH HEALTH SCIENTIST
JESSE BROWN VA MEDICAL CENTER
ASSOC PROF IN PHYSICAL MEDICINE AND REHAB
NORTHWESTERN UNIVERSITY
PROTHETICS ORTHOTICS CENTER
CHICAGO, IL 60611

GUGLIUCCI, MARILYN R, PHD *
PROFESSOR & DIRECTOR, GERIATRICS RESEARCH
DIVISION OF GERIATRICS
UNIVERSITY OF NEW ENGLAND
COLLEGE OF OSTEOPATHIC MEDICINE
BIDDEFORD, ME 04005

HEADLEY, SAMUEL A, PHD
PROFESSOR AND DIRECTOR OF CLINICAL EXERCISE PHYSIOLOGY
DEPT OF EXERCISE SCIENCE AND ATHLETIC TRAINING
SPRINGFIELD COLLEGE
SPRINGFIELD, MA 01109

KIMBREL, NATHAN A., PHD *
CO-DIRECTOR, CLINICAL CORE, MID-ATLANTIC MIRECC
DURHAM VA HEALTH CARE SYSTEM
ASSOCIATE PROFESSOR, PSYCHIATRY AND BEHAVIORAL SCI
SCHOOL OF MEDICINE
DUKE UNIVERSITY
DURHAM, NC 27705

KUNISAKI, KEN M., MD
STAFF PHYSICIAN & MEDICAL DIRECTOR FOR MINNEAPOLIS VA COPD CASE MANAGEMENT PROGRAM
MINNEAPOLIS VA HEALTH CARE SYSTEM
ASSOCIATE PROFESSOR OF MEDICINE
DEPT OF PULMONARY, CRITICAL CARE AND SLEEP
UNIVERSITY OF MINNESOTA
MINNEAPOLIS, MN 55417

LEE, PEARL G, MD *
GERIATRICIAN
ANN ARBOR VA HEALTHCARE SYSTEM
ASSISTANT PROFESSOR, INTERNAL MEDICINE
UNIVERSITY OF MICHIGAN, GERIATRICS
ANN ARBOR, MI 48109

LOCHER, JULIE L., PHD *
PROFESSOR
DEPARTMENT OF MEDICINE
UNIVERSITY OF ALABAMA, BIRMINGHAM
BIRMINGHAM, AL 35294

MCTEAGUE, LISA M, PHD *
PSYCHOLOGIST
CHARLESTON VA MEDICAL CENTER
ASSISTANT PROFESSOR
DEPARTMENT OF PSYCHIATRY AND BEHAVIORAL SCIENCES
MEDICAL UNIVERSITY OF SOUTH CAROLINA
CHARLESTON, SC 29425

MONTGOMERY, LATRICE, PHD *
RESEARCH ASSISTANT PROFESSOR AND CLINICAL PSYCHOLOGIST
ADDICTION SCIENCES DIVISION
DEPARTMENT OF PSYCHIATRY
AND BEHAVIORAL NEUROSCIENCE
UNIVERSITY OF CINCINNATI COLLEGE OF MEDICINE
CINCINNATI, OH 45229

TESKE, JENNIFER ANN, PHD
ASSISTANT PROFESSOR
DEPARTMENT OF NUTRITIONAL SCIENCES
THE UNIVERSITY OF ARIZONA
TUCSON, AZ 85721

WAID-EBBS, JULIA KAY, PHD *
HEALTH SCIENCE SPECIALIST, RR&D BRAIN REHABILITATION RESEARCH CENTER (BRRC)
MALCOM RANDALL VA MEDICAL CENTER
GAINESVILLE, FL 32608

WALLIN, MITCHELL TAYLOR, MD *
CLINICAL ASSOCIATE DIRECTOR
BALTIMORE VA MEDICAL CENTER
ADJUNCT ASSOCIATE PROFESSOR
SCHOOL OF MEDICINE
UNIVERSITY OF MARYLAND
BALTIMORE, MD 21201

YALCH, MATTHEW, PHD *
ASSISTANT PROFESSOR
DEPARTMENT OF CLINICAL PSYCHOLOGY
PALO ALTO UNIVERSITY
PALO ALTO, CA 94304

EXECUTIVE SECRETARY

BOUWER, H. G. ARCHIE, PHD
DEPUTY ASSOCIATE CHIEF OF STAFF
PORTLAND VA HEALTH CARE SYSTEM
SOM-MOLECULAR MICROBIOLOGY & IMMUNOLOGY DEPT
OREGON HEALTH SCIENCES UNIVERSITY
PORTLAND, OR 97213

SCIENTIFIC REVIEW OFFICER

GROER, SHIRLEY, PHD
SCIENTIFIC PROGRAM MANAGER
DEPARTMENT OF VETERANS AFFAIRS
OFFICE OF RESEARCH AND DEVELOPMENT
REHABILITATION RESEARCH AND DEVELOPMENT SERVICE
WASHINGTON, DC 20420

* Temporary Member. For grant applications, temporary members may participate in the entire meeting or may review only selected applications as needed.

Consultants are required to absent themselves from the room during the review of any application if their presence would constitute or appear to constitute a conflict of interest.
